# Supplementary figures and images for: Acute bee paralysis virus regulation of microRNA expression dynamics in the insect host (Apis mellifera) cell line, AmE-711
Source: Front Cell Infect Microbiol. 2026 Jan 5;15:1709271. doi: 10.3389/fcimb.2025.1709271 (PMC12812641; doi:10.3389/fcimb.2025.1709271)

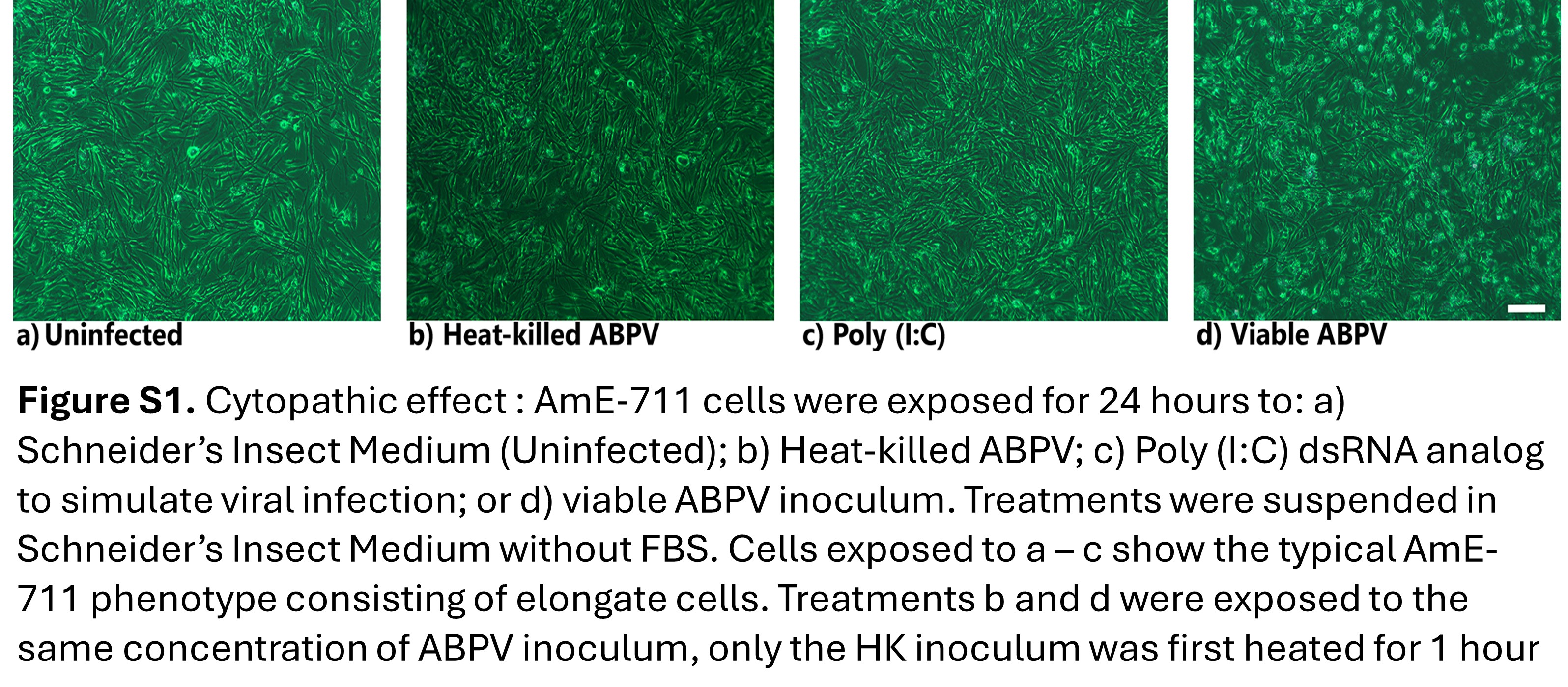

Supplement: Supplementary file 1 [file Image1.jpeg]

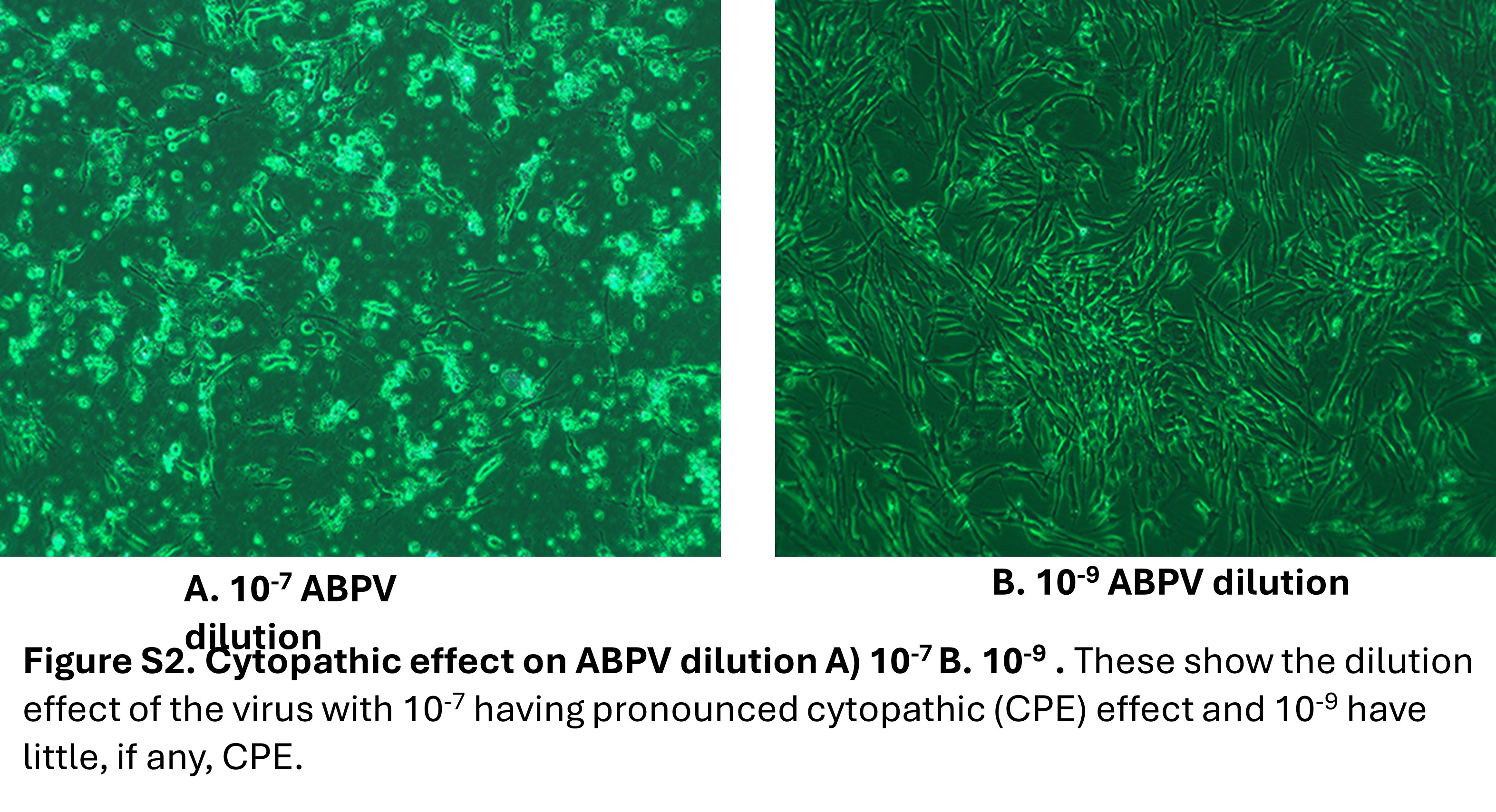

Supplement: Supplementary file 2 [file Image2.jpeg]
